# Gene expression and functional abnormalities in XX/*Sry* Leydig cells

**DOI:** 10.1038/s41598-020-80741-z

**Published:** 2021-01-12

**Authors:** Shogo Yanai, Takashi Baba, Kai Inui, Kanako Miyabayashi, Soyun Han, Miki Inoue, Fumiya Takahashi, Yoshiakira Kanai, Yasuyuki Ohkawa, Man Ho Choi, Ken-ichirou Morohashi

**Affiliations:** 1 Department of Systems Life Sciences, Graduate School of Systems Life Sciences, Kyushu University, Maidashi 3-1-1, Higashi-ku, Fukuoka, 812-8582 Japan; 2 Department of Molecular Biology, Graduate School of Medical Sciences, Kyushu University, Maidashi 3-1-1, Higashi-ku, Fukuoka, 812-8582 Japan; 3 Molecular Recognition Research Center, Korea Institute of Science and Technology, Seoul, 02792 Korea; 4 Department of Veterinary Anatomy, The University of Tokyo, Yayoi 1-1-1, Bunkyo-ku, Tokyo, 113-8657 Japan; 5 Division of Transcriptomics, Medical Institute of Bioregulation, Kyushu University, Maidashi 3-1-1, Higashi-ku, Fukuoka, 812-8582 Japan; 6 AMED-CREST, Japan Agency for Medical Research and Development, Maidashi 3-1-1, Higashi-ku, Fukuoka, 812-8582 Japan

**Keywords:** Sterols, Transcriptomics

## Abstract

The *SRY* gene induces testis development even in XX individuals. However, XX/*Sry* testes fail to produce mature sperm, due to the absence of Y chromosome carrying genes essential for spermatogenesis. XX/*Sry* Sertoli cells show abnormalities in the production of lactate and cholesterol required for germ cell development. Leydig cells are essential for male functions through testosterone production. However, whether XX/*Sry* adult Leydig cells (XX/*Sry* ALCs) function normally remains unclear. In this study, the transcriptomes from XY and XX/*Sry* ALCs demonstrated that immediate early and cholesterogenic gene expressions differed between these cells. Interestingly, cholesterogenic genes were upregulated in XX/*Sry* ALCs, although downregulated in XX/*Sry* Sertoli cells. Among the steroidogenic enzymes, CYP17A1 mediates steroid 17α-hydroxylation and 17,20-lyase reaction, necessary for testosterone production. In XX/*Sry* ALCs, the latter reaction was selectively decreased. The defects in XX/*Sry* ALCs, together with those in the germ and Sertoli cells, might explain the infertility of XX/*Sry* testes.

## Introduction

It has been established that the *SRY* (sex-determining region on the Y chromosome) gene is responsible for the differentiation of the testes in mammals^1,2^. Indeed, injection of the *Sry* gene into fertilized XX mouse eggs leads to testis development in XX fetuses. However, XX mice carrying the *Sry* transgene (XX/*Sry* mice) suffer from spermatogenic failure^3,4^. Although the developmental defects of germ cells have been thought to be caused by the lack of Y-chromosome genes essential for spermatogenesis^5^, the reason for this infertility in XX/*Sry* mice is still under discussion. In fact, our previous study identified disfunction of XX/*Sry* Sertoli cells^6^. In general, Sertoli cells support the differentiation of germ cells by providing them with nutrients including lactate^7^ and cholesterol^8^. XX/*Sry* Sertoli cells were found to synthesize these substances less than XY Sertoli cells, due to lower expression levels of the genes required for their synthesis^6^.

In addition to Sertoli cells, testes contain Leydig cells, which are developmentally divided into two types, fetal-type (FLCs) and adult-type (ALCs). During the fetal stage, FLCs emerge within the interstitial space of the fetal testes and increase in number during embryonic development. After birth, FLCs are gradually substituted with ALCs^9^. Finally, in the adult stage, the testicular interstitial space is predominantly occupied by ALCs, although a small population of FLCs remains^9–11^. With respect to the Leydig cells in XX/*Sry* testes, it remains largely unclear whether the ALCs in XX/*Sry* mice exhibit functions equivalent to XY ALCs.

In general, ALCs are characterized by the functional capacity to produce testosterone. Four enzymes have been implicated in the synthesis of testosterone from cholesterol: cytochrome P450 family members cholesterol side-chain cleavage enzyme (CYP11A1) and 17α-hydroxylase/17,20-lyase (CYP17A1); 3β-hydroxysteroid dehydrogenase (HSD3B1 and HSD3B6); and 17β-hydroxysteroid dehydrogenase (HSD17B3)^12,13^. Of these enzymes, CYP17A1 uniquely mediates two distinct reactions: 17α-hydroxylation and C17,20-cleavage of steroids^14^. Both reactions are successively mediated by CYP17A1 in the Leydig cells of all mammalian species.

Ad4BP/SF-1 (adrenal-4 binding protein/steroidogenic factor 1/NR5A1^15^) was initially identified as a nuclear receptor-type transcription factor that regulates the gene transcription of *CYP11A1* and *CYP11B1* (steroid 11β-hydroxylase)^16–18^. Thereafter, many studies have investigated whether other steroidogenic genes are also regulated by Ad4BP/SF-1. These studies identified *HSD3B2*^19,20^, *CYP17A1*^21–23^, and *CYP19A1*^24^ as target genes of this factor. Thus, it has been widely accepted that Ad4BP/SF-1 plays a central role in the regulation of steroidogenic genes^25,26^.

All steroid hormones are synthesized from cholesterol. In addition to special usage for steroidogenesis, cholesterol is known to be an essential component of various cellular membranes^27^. In accordance with this broad range of requirements for cholesterol, cholesterogenic genes are expressed in a variety of cell types. Extensive investigation of cholesterogenic gene regulation in the liver has led to the identification of sterol regulatory element binding protein 2 (SREBP2, encoded by *SREBF2*) as the key transcription factor regulating all cholesterogenic genes^28^. In addition to this key molecule, Ad4BP/SF-1 has recently been shown to be involved in cholesterogenic gene regulation in steroidogenic cells^29^.

In this study, we investigated whether XX/*Sry* ALCs are functionally different from XY ALCs. Comparison of the transcriptomes obtained from these two types of cells revealed that the expression of immediate early genes and cholesterogenic genes was altered in the XX/*Sry* ALCs. In addition, we found that the 17,20-lyase reaction mediated by CYP17A1 was specifically affected in XX/*Sry* ALCs.

## Results

### Increase of ALCs in XX/Sry testes

**Figure 1:**
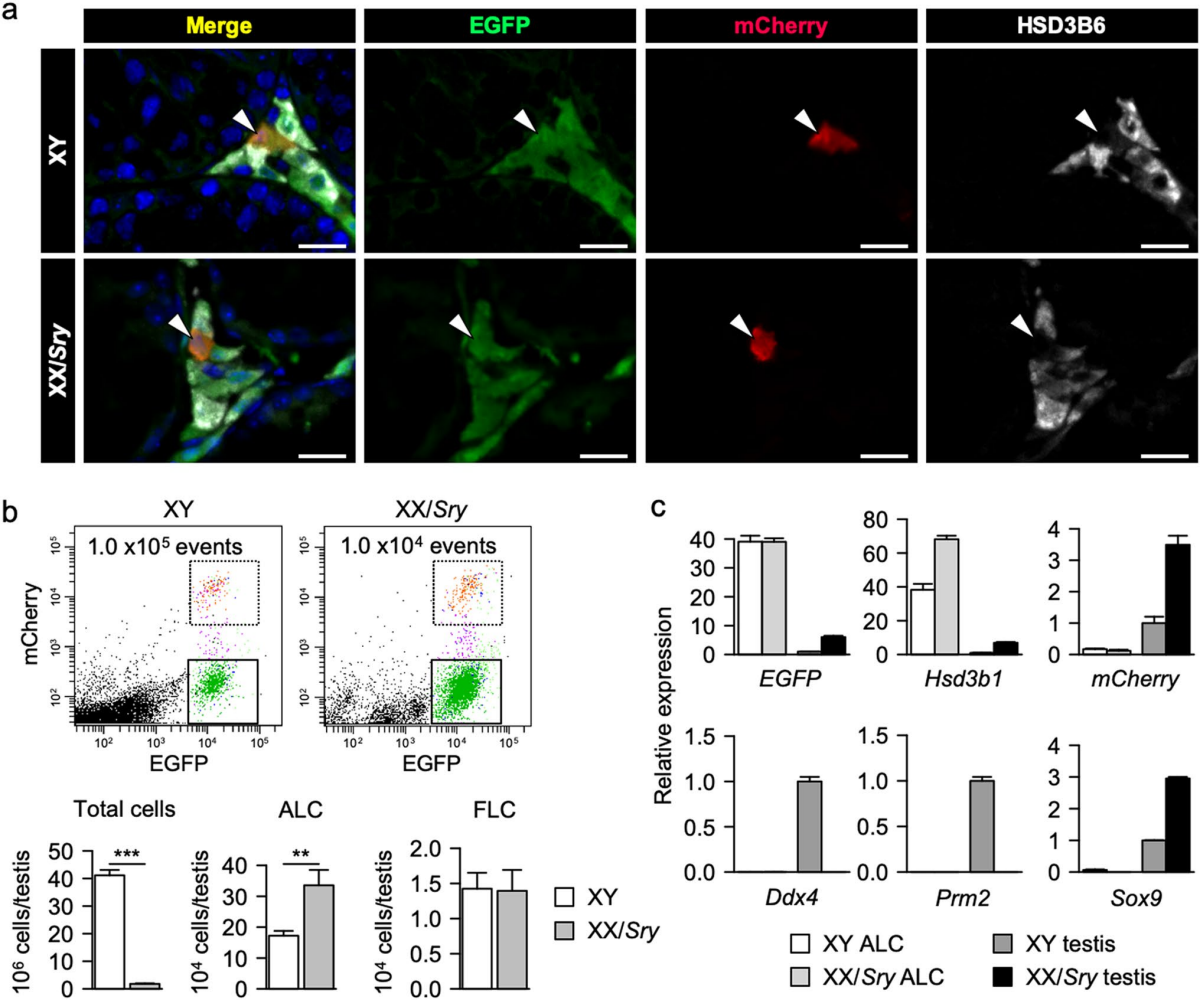
Preparation of ALCs from XY and XX/*Sry* testes. (**a**) XY and XX/*Sry* testes from eight-week-old *Ad4BP-BAC-EGFP/mFLE-mCherry* mice were immunostained with antibodies for EGFP (green), mCherry (red), and HSD3B6 (white). Nuclei were stained with DAPI (blue). Merged images are shown on the left. Arrowheads indicate FLCs (EGFP/mCherry double-positive, HSD3B6-negative). ALCs were detected as EGFP/HSD3B6 double-positive, mCherry-negative cells. Scale bars = 20 μm. (**b**) Whole cell preparations from the testes of 8-week-old XY and XX/*Sry* mice were analyzed via FACS. The cells surrounded by solid lines were recovered as EGFP single-positive ALCs, whereas those surrounded by dotted lines were recovered as EGFP/mCherry double-positive FLCs (upper panels). Total testicular cells were counted. Numbers of ALCs and FLCs per testis are calculated using the results obtained by FACS. Nine biologically independent samples (n = 9) were used for counting (lower panels). (**c**) The expression levels of marker genes in the sorted XY and XX/*Sry* ALCs, along with those of *EGFP* and *mCherry,* were examined using qRT-PCR. XY and XX/*Sry* testes were used as controls. Three biologically independent samples (n = 3) were used. The data were normalized to *Actb* and are presented as means ± SEM. * *p* < 0.05, ** *p* < 0.01, *** *p* < 0.001, using Student’s *t*-test (**b**). R software version 3.4.3 (https://www.r-project.org) was used to draw the plots in (**b**,**c**).

It was previously believed that FLCs are completely replaced by ALCs after birth. However, our previous studies have demonstrated that FLCs persist in adult mouse testes^11,30^. Therefore, to selectively investigate ALCs, we established a mouse line carrying *Ad4BP-BAC-EGFP* and *mFLE-mCherry* as transgenes. In the mouse testes, FLCs were labeled with both EGFP and mCherry, whereas ALCs were labeled with EGFP alone. This double transgenic mouse line thus enabled us to isolate ALCs and FLCs with no mutual contamination. We transferred these two transgenes into XY and XX/*Sry* mice to obtain XY and XX/*Sry* ALCs as EGFP single-positive cells. As shown in Fig. 1a, we found both EGFP single-positive and EGFP/mCherry double-positive Leydig cells in both XY and XX/*Sry* testes. HSD3B6, an ALC marker, was colocalized with the EGFP in the single-positive Leydig cells, indicating that these cells were ALCs.

Fluorescence-activated cell sorting (FACS) of the testicular cells enabled us to isolate two distinct Leydig cell populations, EGFP single-positive ALCs and EGFP/mCherry double-positive FLCs, from both XY and XX/*Sry* testes (Fig. 1b). Since the XX/*Sry* adult testes were hypoplastic and lacked all germ cells (Supplemental Fig. 1), the total number of cells in a single XX/*Sry* testis was substantially lower than that in a single XY testis. Surprisingly, however, the number of ALCs in the XX/*Sry* testis was close to double that in the XY testis (Fig. 1b). The purity of the ALC fraction prepared by FACS was examined using qRT-PCR for testicular cell marker genes. *EGFP* and *Hsd3b1* were highly enriched in ALCs from both XY and XX/*Sry* testes, whereas *mCherry* was barely detectable in either group (Fig. 1c). Germ cell markers *Ddx4* and *Prm2* were undetectable in the ALCs, as was Sertoli cell marker *Sox9*. These results indicate that the ALC fraction used in this study was unlikely to have been contaminated with FLCs, germ cells, or Sertoli cells.

### Differential gene expression between XY and XX/Sry ALCs

**Figure 2:**
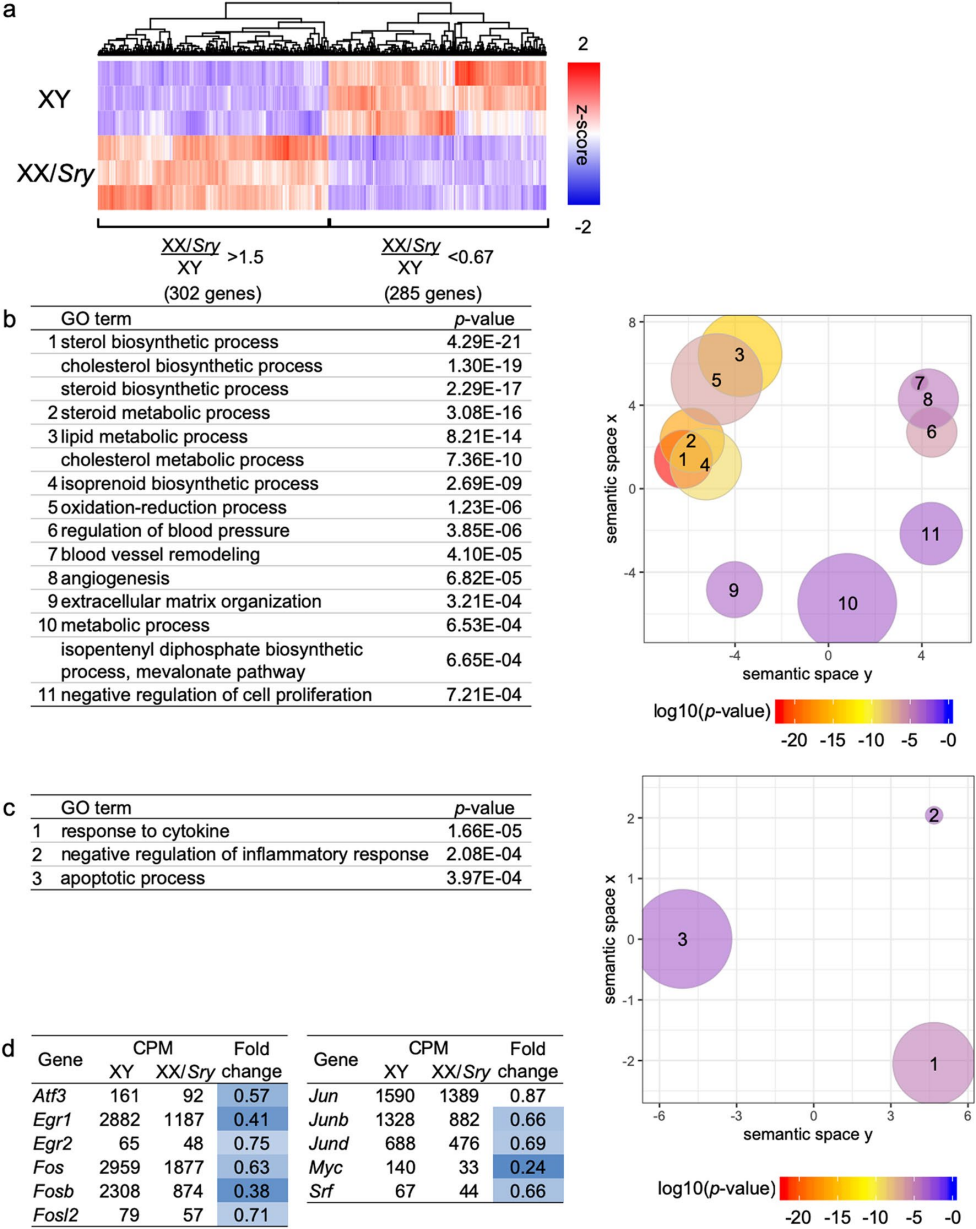
Genes differentially expressed in XY and XX/*Sry* ALCs. (**a**) Heatmap of differentially expressed genes in XY and XX/*Sry* ALCs, based on a comparison of their transcriptomes. The expression levels of 302 genes were at least 1.5 times higher in XX/*Sry* than XY ALCs, and those of 285 genes in XX/*Sry* were at most two third of those in XY ALCs. (**b**) GO terms identified by GO pathway analyses of the 302 upregulated genes in XX/*Sry* ALCs are indicated (left) and visualized in a two-dimensional plot using REVIGO (right). The top 15 GO terms (*p* < 0.001) were plotted by REVIGO after four redundant terms had been excluded. The numbers in the plots correspond to the numbers of the GO terms shown in the left-hand column of the table. The plot colors indicate the *p*-values of the GO terms as per the table, and plot sizes indicate the specificity of the terms (plots for more specific terms are smaller). (**c**) GO terms identified by GO pathway analyses of the 285 genes downregulated in XX/*Sry* ALCs are indicated and visualized as described above. The top three GO terms (*p* < 0.001), which were used for the analysis, are listed in the table on the left and plotted in the panel on the right. (**d**) Expression of immediate early genes in XY and XX/*Sry* ALCs. CPMs are means of biological triplicates for each cell type. Cells in the fold change column are shaded according to difference in expression: the greater the decrease, the deeper the blue shading. R software version 3.4.3 (https://www.r-project.org) was used to draw the plots in (**a**–**c**).

To investigate whether XX/*Sry* ALCs differ from XY ALCs, transcriptomes were obtained from three biologically independent sets of ALC samples each from XY and XX/*Sry* testes. Considering the high unique mapping rate of the sequence reads (approximately 90%) and the high reproducibility between the biological triplicates (correlation coefficient >  = 0.992; Supplemental Fig. 2a and 2b), the quality of the transcriptome datasets was considered sufficient for further examination. Comparison of the transcriptomes revealed that the expression levels of 302 and 285 genes were more than 1.5-fold higher and lower, respectively, in the XX/*Sry* ALCs compared to the XY ALCs (Fig. 2a, Supplemental Tables 1 and 2).

These differentially expressed genes were subjected to GO pathway analysis to investigate which biological processes are associated with the genes up- and downregulated in the XX/*Sry* ALCs. As listed in Fig. 2b (left panel), ‘sterol biosynthetic process’, ‘cholesterol biosynthetic process’, ‘steroid biosynthetic process’, and ‘steroid metabolic process’ were strongly related to the genes upregulated in the XX/*Sry* ALCs. Of these genes, the ones commonly associated with these processes were predominantly cholesterogenic. The next most strongly represented process was ‘lipid metabolic process’. Although the gene list for this process includes cholesterogenic genes, it also includes genes specifically required for lipid synthesis. In accordance with the sharing of cholesterogenic genes, REVIGO plot analysis suggested that these processes involving cholesterogenesis seemed to form a cluster at the top left (right panel in Fig. 2b).

Multiple terms related to blood vessels were listed, and these formed another cluster (Fig. 2b). This suggests that although we could not find any clear defect, the blood vessels of the XX/*Sry* testes may be affected by the differential expression of these genes. In addition to the genes included in the terms above, we noticed that the expression of extracellular matrix genes (such as those associated with several types of collagen, laminin, and biglycan) was higher in the XX/*Sry* ALCs, suggesting that the extracellular matrix surrounding XX/*Sry* ALCs is different from that surrounding XY ALCs.

A few biological processes were related to the genes downregulated in the XX/*Sry* ALCs, and their *p*-values were relatively large compared with those related to the upregulated genes (left panel in Fig. 2c). REVIGO plot analysis suggested that these biological terms were not closely related (right panel in Fig. 2c). Although any close correlations between the listed terms and Leydig cell functions were unlikely, we noticed that the term ‘response to cytokine’ includes the *Fos*, *Junb*, and *Jund* genes. These gene products, leucine zipper-type transcription factors, have been studied extensively and found to be activated in response to a variety of stimuli, such as serum, growth factors, and cytokines^31^. Since these genes have been classified into immediate early genes, we examined whether the expression levels of other genes in this group were affected in XX/*Sry* ALCs. Interestingly, many other immediate early genes, such as *Atf3*, *Egr1*, and *Myc*, were also downregulated in XX/*Sry* ALCs compared to XY ALCs (Fig. 2d).

### Cholesterogenic gene expression increased in XX/Sry ALCs

**Figure 3:**
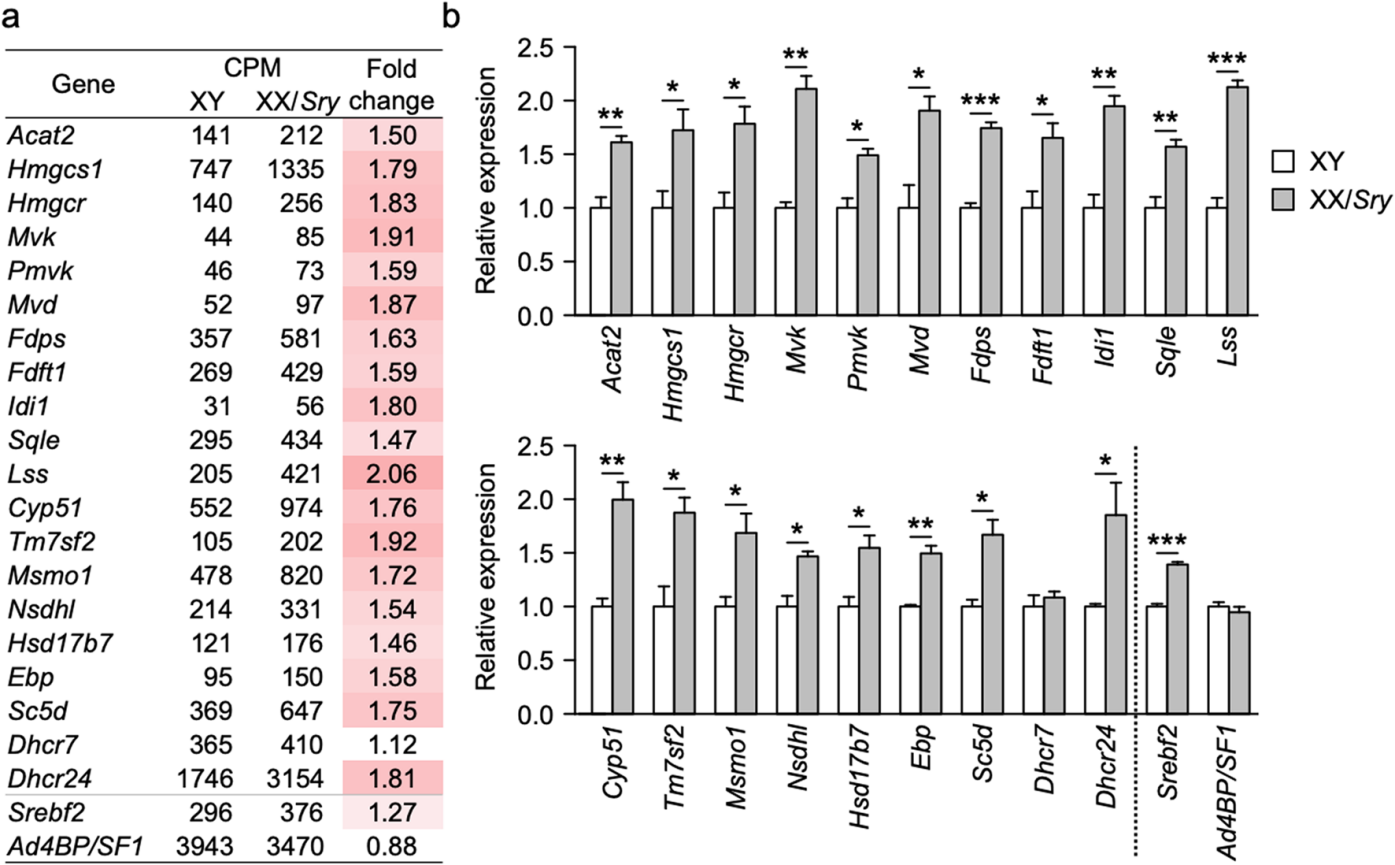
Cholesterogenic gene expression increased in XX/*Sry* ALCs. (**a**) Expression of cholesterogenic genes as well as *Srebf2* and *Ad4BP/SF-1* was extracted from the transcriptome datasets for the XY and XX/*Sry* ALCs. CPMs are means of biological triplicates. Cells in the fold change column are shaded according to difference in expression: the greater the increase, the deeper the red shading. (**b**) Expression of the cholesterogenic genes, *Srebf2*, and *Ad4BP/SF-1* was validated using qRT-PCR. The data were normalized to *Actb* and are presented as means ± SEM. Three biologically independent samples (n = 3) were used. *p < 0.05, **p < 0.01, ***p < 0.001, using Student’s *t*-test (**b**). R software version 3.4.3 (https://www.r-project.org) was used to draw the plots in (**b**).

Since cholesterogenic pathway is involved in the biological functions activated in the XX/*Sry* ALCs, we examined the expression of cholesterogenic genes in the XY and XX/*Sry* ALCs. The transcriptome data indicated that almost all the cholesterogenic genes were upregulated more than 1.5-fold in the XX/*Sry* ALCs (Fig. 3a). This increased expression was confirmed by qRT-PCR (Fig. 3b). Numerous studies have demonstrated that SREBP2, encoded by *Srebf2*, plays a crucial role in cholesterogenic gene regulation^28^. In fact, it has been demonstrated that SREBP2 accumulates in the regions upstream of cholesterogenic genes^32^. In addition, we recently demonstrated that Ad4BP/SF-1 also accumulates at cholesterogenic gene loci in steroidogenic cells, including ALCs^29^. Therefore, we expected that at least one of these two transcription factors would also be upregulated in the XX/*Sry* ALCs. Although the expression of *Ad4BP/SF-1* was unaltered in these cells, *Srebf2* expression was slightly higher in the XX/*Sry* ALCs. This altered expression of *Srebf2* could be responsible, at least in part, for the observed enhanced expression of cholesterogenic genes in the XX/*Sry* ALCs.

### Differential effects on gene expression between XX/Sry ALCs and Sertoli cells

**Figure 4:**
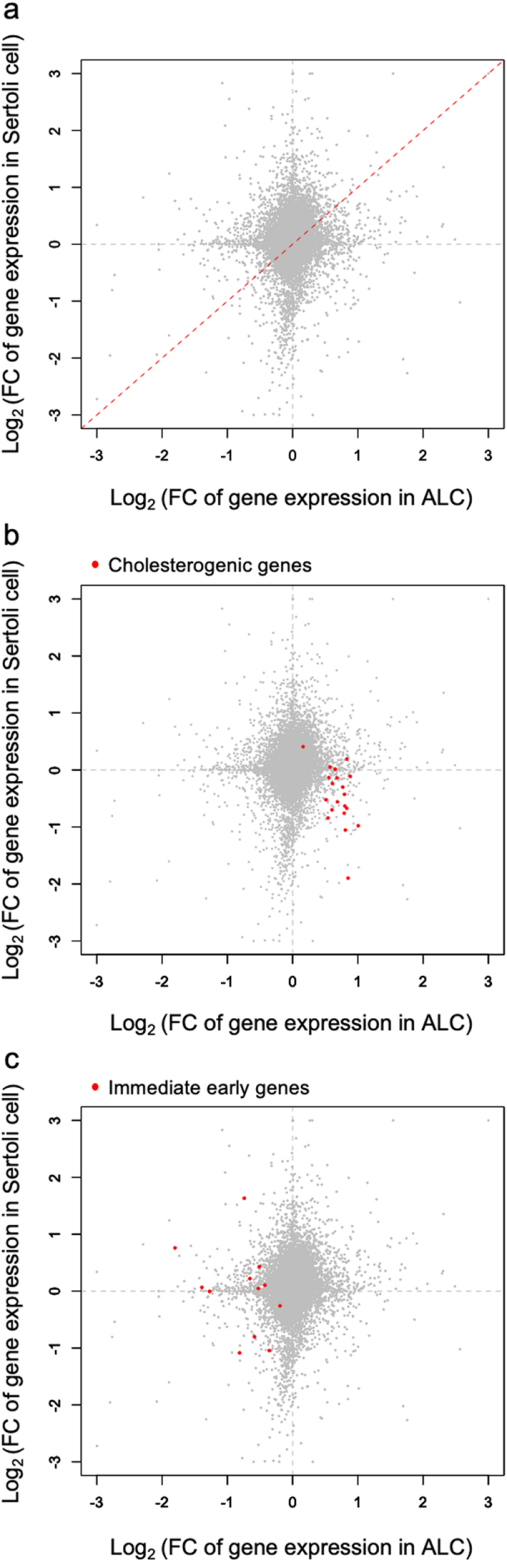
Differentially affected gene expression in XX/*Sry* ALCs and Sertoli cells. (**a**–**c**) Fold changes in gene expression were calculated as the ratio of the CPM values for XX/*Sry* Sertoli cells to those for XY Sertoli cells, and of CPM values for XX/*Sry* ALCs to those for XY ALCs. (**a**) Whole genes were plotted according to the calculated values. The dotted red line indicates where genes that are similarly expressed in the two cell types would fall. The horizontal and vertical axes represent log_2_ fold change (FC) in gene expression in the ALCs and Sertoli cells, respectively. A dot corresponds to a single gene. (**b**) Cholesterogenic genes are depicted as red dots. (**c**) Immediate early genes are depicted as red dots. R software version 3.4.3 (https://www.r-project.org) was used to draw the plots in (**a**–**c**).

We previously compared gene expression between XY and XX/*Sry* Sertoli cells and found that cholesterogenic genes were downregulated in the latter^6^. Accordingly, the present study showed that cholesterogenic gene expression was affected in opposite ways between the XX/*Sry* ALCs and Sertoli cells. We graphically compared whole-gene expression changes in the two types of cells. Fold changes in gene expression levels (XX/*Sry* vs. XY) for Sertoli cells were plotted on the vertical axis and for ALCs on the horizontal axis (Fig. 4). If genes were up- or downregulated in both types of XX/*Sry* cells, they would be lie on or near the red broken line in Fig. 4a. However, there was no particular pattern of distribution along this line. Instead, a considerable number of genes were aligned along the lines x = 0 or y = 0, suggesting that the alteration of gene expression was probably cell-type specific.

Cholesterogenic genes are indicated with red dots in the plot shown in Fig. 4b. As expected, many of these genes are localized in the lower right quadrant, which is consistent with our finding that cholesterogenic genes were upregulated in the XX/*Sry* ALCs but downregulated in the XX/*Sry* Sertoli cells. As mentioned above, immediate early genes were downregulated in the XX/*Sry* ALCs. However, no biased expression of this group was detected in the XX/*Sry* Sertoli cells. Consistent with this, the immediate early genes are distributed within the left half of the plot area (Fig. 4c).

### Altered expression of genes normally enriched in ALCs

**Figure 5:**
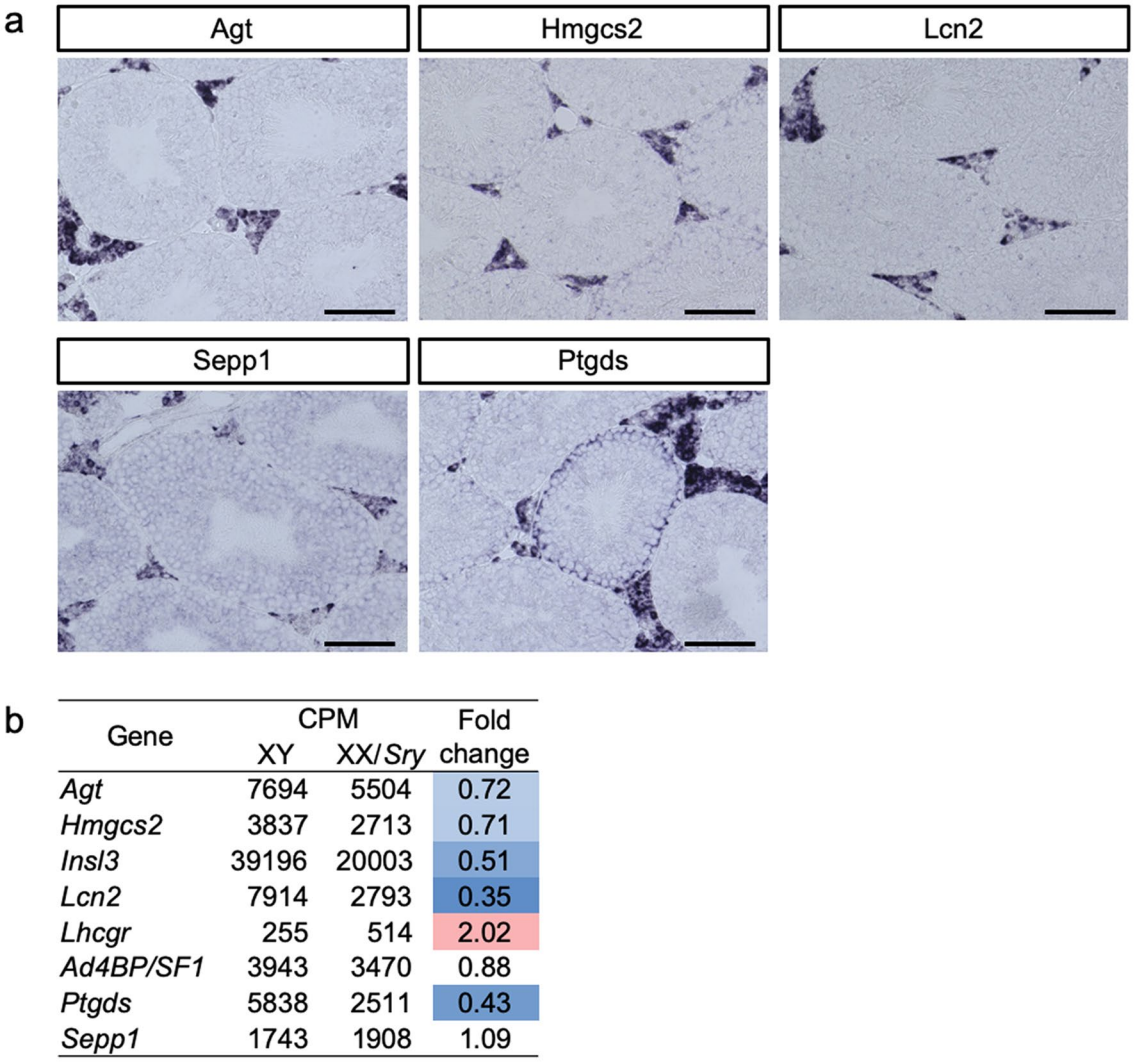
Altered expression of genes normally enriched in ALCs. (**a**) Expression of *Agt*, *Hmgcs2*, *Lcn2*, *Sepp1* and *Ptgds* in XY adult testes was examined using in situ hybridization. Scale bars = 100 μm. (**b**) Expression of genes that are normally enriched in ALCs in XY and XX/*Sry* ALCs was obtained from the transcriptome datasets. The displayed CPMs are the means of biological triplicates. Increased and decreased gene expression in the XX/*Sry* ALCs is indicated in red and blue, respectively, with deeper shading for larger differences.

It has been established that the expression levels of *Insl3*, *Ad4BP/SF-1*, and *Lhcgr* (Luteinizing hormone/choriogonadotropin receptor) are enriched in ALCs^33,34^. In addition, we previously found several candidate genes that are probably enriched in ALCs by comparing the transcriptomes of ALCs and FLCs^30^. In the present study, we examined the expression of these genes via in situ hybridization. As shown in Fig. 5a, *Agt* (angiotensinogen) was expressed in ALCs but not in Sertoli or germ cells in adult testes. Enriched expression in ALCs has previously been observed for *Hmgcs2* (3-hydroxy-3-methylglutaryl-CoA synthase 2)^35,36^, *Lcn2* (lipocalin-2)^37^, and *Sepp1* (selenoprotein P, plasma, 1)^38^. A high level of *Ptgds* (prostaglandin D2 synthase) expression was detected in ALCs, although the expression was also detected in Sertoli cells from some, but not all, testicular tubules^39^. Interestingly, the transcriptomes obtained in the present study revealed that many of these genes were downregulated in the XX/*Sry* ALCs (Fig. 5b).

### Steroidogenesis possibly affected by decreased steroid 17,20-lyase activity

**Figure 6:**
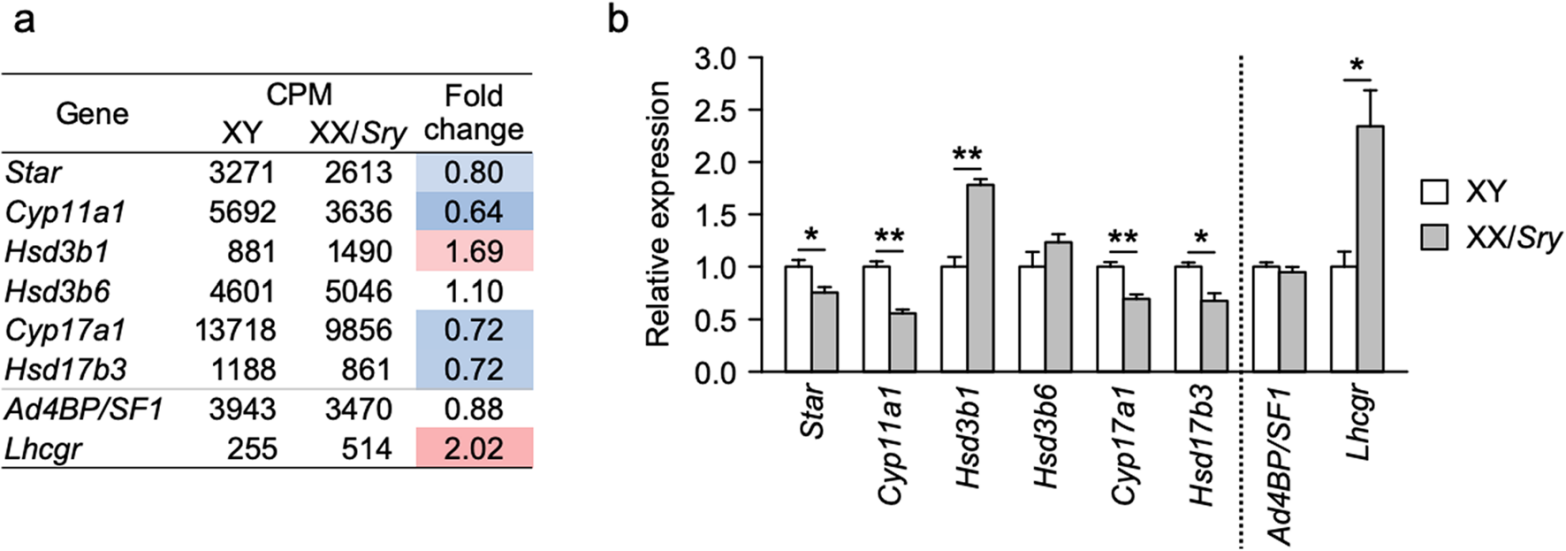
Steroidogenic gene expression is affected in XX/*Sry* ALCs. (**a**) Expression of steroidogenic genes as well as *Ad4BP/SF-1* and *Lhcgr* in the XY and XX/*Sry* ALC transcriptome datasets. CPMs are the means of biological triplicates. Increased and decreased gene expression in the XX/*Sry* ALCs is indicated in red and blue, respectively, with deeper shading for larger differences. (**b**) Expression of the genes shown in (**a**) was validated by qRT-PCR. The data were normalized to *Actb* and are presented as means ± SEM. Three biologically independent samples (n = 3) were used. * *p* < 0.05, ** *p* < 0.01, *** *p* < 0.001, using Student’s *t*-test (**b**). R software version 3.4.3 (https://www.r-project.org) was used to draw the plots in (**b**).

We previously demonstrated that the amount of testosterone synthesized in XX/*Sry* testes at postnatal day 21 was smaller than in XY testes^6^. We therefore extracted the expression data for steroidogenic genes from our transcriptome datasets. The expression of *Star*, *Cyp11a1*, *Cyp17a1*, and *Hsd17b3* was decreased to approximately 70% of XY ALC levels in the XX/*Sry* ALCs (Fig. 6a). Similar expression profiles for these genes were obtained using qRT-PCR (Fig. 6b). The expression of *Ad4BP/SF-1*, a key regulator of steroidogenic gene expression, was not significantly affected in the XX/*Sry* ALCs, while that of *Lhcgr* was more than doubled.

**Figure 7:**
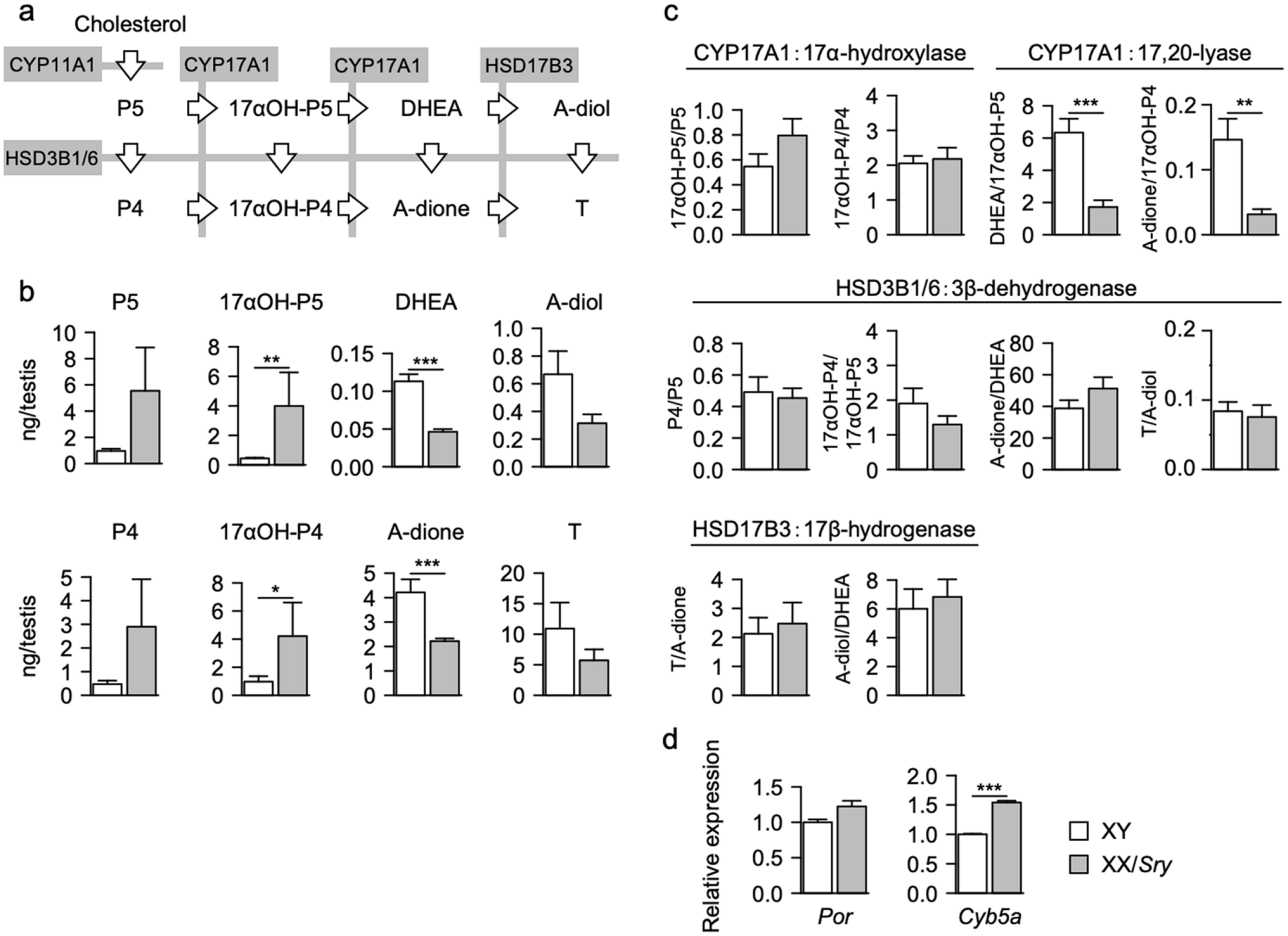
17,20-lyase activity of CYP17A1 is reduced in XX/*Sry* testes. (**a**) The pathway of testosterone synthesis from cholesterol. The enzymes implicated in the pathway are shown in gray boxes. P5, pregnenolone; P4, progesterone; 17αOH-P5, 17α-hydroxy-pregnenolone; 17αOH-P4, 17α-hydroxyprogesterone; DHEA, dehydroepiandrosterone; A-dione, androstenedione; A-diol, androstenediol; T, testosterone. (**b**) The quantities of these steroids in the XY and XX/*Sry* testes were determined using GC–MS/MS. Eight biologically independent samples (n = 8) were used in this analysis. (**c**) Metabolic ratios for all reactions were calculated as the ratio of substrate to metabolite using the quantities of intratesticular steroids detected in the XY and XX/*Sry* testes. (**d**) Expression of *Por* and *Cyb5a* genes were examined using qRT-PCR. The data were normalized to *Actb* and are presented as means ± SEM. Three biologically independent samples (n = 3) were used for each cell type. * *p* < 0.05, ** *p* < 0.01, *** *p* < 0.001, using Mann–Whitney *U* test (**b,c**) and Student’s t-test (**d**). R software version 3.4.3 (https://www.r-project.org) was used to draw the plots in (**b–d**).

To examine whether these changes affected steroidogenesis, the quantities of steroidal molecules were determined for both XY and XX/*Sry* testes. Testosterone synthesis from cholesterol is mediated by multiple enzymes (Fig. 7a). As indicated in Fig. 7b, the quantities of P5 (pregnenolone), P4 (progesterone), 17αOH-P5 (17α-hydroxypregnenolone), and 17αOH-P4 (17α-hydroxyprogesterone) in the XX/*Sry* testes were greater than those in the XY testes. Interestingly, however, the quantities of DHEA (dehydroepiandrosterone), A-dione (androstenedione), A-diol (androstenediol), and T (testosterone) in the XX/*Sry* testes were smaller than those in the XY testes. Based on these steroid quantities, the enzymatic activities were evaluated by calculating metabolic ratios. While 17α-hydroxylation, 3β-dehydrogenation, and 17β-hydroxylation were not significantly altered, the 17,20-lyase reaction was substantially reduced in the XX/*Sry* testes (Fig. 7c). Interestingly, 17α-hydroxylation and 17,20-lyase reaction are mediated by a single enzyme, CYP17A1. Electrons from NADPH/NADH required for these reactions are transferred to CYP17A1 from POR (P450 oxidoreductase) and/or CYB5A (cytochrome b5a). The expression of *Cyb5a* was increased in the XX/*Sry* ALCs (Fig. 7d), but that of *Por* was not significantly altered.

## Discussion

In the present study, we aimed to determine whether XX/*Sry* ALCs are functionally equivalent to XY ALCs. To investigate it, transcriptomes obtained from XY and XX/*Sry* ALCs were compared. As the consequence, the expression of 302 and 285 genes was found to be up- and downregulated, respectively, in the XX/*Sry* ALCs compared to XY ALCs. These gene sets suggested that several biological activities and processes are affected in XX/*Sry* ALCs.

There are potential reasons why the number of ALCs was increased in XX/*Sry* testes. LH has been established to be one of the key molecules for differentiation of ALCs. In fact, ALCs were decreased in the testis of *Lhcgr* KO mice^40^. Moreover, transgenic overexpression of human chorionic gonadotropin (*HCG*), which potentially binds and activates LHCGR, resulted in an increase of ALCs^41,42^. Based on these findings, the increase of ALCs in the XX/*Sry* testes might be due to the increased expression of *Lhcgr*.

In addition to the endocrine factor above, there are several paracrine factors regulating differentiation of ALCs. Desert hedgehog (DHH), secreted by Sertoli cells, stimulates proliferation of stem Leydig cells and their differentiation into ALCs^43^. However, our previous study showed that the expression of *Dhh* was not altered in the XX/*Sry* Sertoli cells compared to XY cells^6^ (Supplemental Fig. 3a). Likewise, the expression of the hedgehog signaling components such as *Ptch1/2* and *Smo* was not affected in the XX/*Sry* ALCs (Supplemental Fig. 3b). PDGF is another factor to activate proliferation of stem Leydig cells^43^. Although it has been unclear which cells synthesize PDGFs in adult testes, the expression of *Pdgfc* was increased in the XX/*Sry* Sertoli cells. Interestingly, the expression of the receptor gene, *Pdgfra*, was increased in the XX/*Sry* ALCs. Taken together, it was suggested that the increase of ALCs in the XX/*Sry* testes might be attributable to the augmentation of PDGF together with LH signaling.

ALCs actively synthesize testosterone through abundant expression of steroidogenic genes. Our transcriptomic analysis revealed that the expression of all steroidogenic genes except *Hsd3b1* and *Hsd3b6* was lower in the XX/*Sry* ALCs compared to XY ALCs. Similarly, we found that the expression of genes normally enriched in ALCs was suppressed, suggesting that the characteristic features of ALCs were affected in the XX/*Sry* ALCs. With respect to the reason for the suppressed expression of these genes, it is interesting to note the downregulation of immediate early genes, whose expression is activated by multiple stimuli^31^, in the XX/*Sry* ALCs. Indeed, the immediate early genes such as *Fos*, *Jun*, *Junb*, and *Jund* (AP1 family members) are activated in ALCs by hCG^44^. It could therefore be assumed that the gene products above activate cellular functions by enhancing the transcription of certain sets of target genes. In fact, steroidogenic gene transcription is regulated by the AP1 family members^45,46^. In addition to the steroidogenic genes, it has been demonstrated that *LCN2* displaying ALC-enriched expression is regulated by EGR1^47^. In the present study, we demonstrated that the expression levels of immediate early genes were decreased in the XX/*Sry* ALCs. The decreased expression of steroidogenic and ALC-enriched genes might therefore be caused by the downregulation of immediate early genes.

Based on this scenario, the concentration of LH secreted by the pituitary and the expression of its receptor, LHCGR, in ALCs should be considered. Our previous study showed that the serum LH concentration in the XX/*Sry* mice was comparable to that of the XY mice^6^, but the present study showed that the expression of *Lhcgr* was higher in the XX/*Sry* ALCs than in the XY ALCs. Therefore, the XX/*Sry* ALCs might receive more effectively the LH signal than the XY ALCs. If it is the case, gene transcription downstream of LH signal such as *Fos* and *Jun* could be activated. Nevertheless, the expression of the immediate early genes was found to be downregulated. Therefore, this inconsistent outcome suggests that intracellular signal transduction might be abnormally regulated in XX/*Sry* ALCs, although it remains unknown which components and/or steps may be affected.

Many transcription factors have been shown to regulate steroidogenic genes. Our transcriptome datasets revealed that the expression of *Cebpb* (*C/EBPβ*) and *Fos* was decreased less than 0.67-fold while that of *Nr3c1* was increased more than 1.5-fold in the XX/*Sry* ALCs (Supplemental Fig. 4). C/EBPβ and FOS were reported to regulate positively mouse *Star* and human *CYP11A1* genes^46,48,49^. Therefore, the decreased expression of *Cyp11a1* and *Star* genes might be due to the downregulated expression of *Cebpb* and *Fos* in the XX/*Sry* ALCs. NR3C1 (GR) was reported to act as a suppressor of mouse *Star* gene transcription^50^. Thus, the upregulated expression of *Nr3c1* might be responsible for the decreased expression of *Star* gene in the XX/*Sry* ALCs.

Steroidogenesis from cholesterol takes place via multiple enzymatic reactions. Based on our analyses of the metabolic ratios, we realized that the 17,20-lyase reaction mediated by CYP17A1 was selectively affected in the XX/*Sry* ALCs. CYP17A1 catalyzes two reactions: 17α-hydroxylation and 17,20-lyase reaction^14^. In many mammalian species, cortisol (glucocorticoid) is synthesized in the zona fasciculata of the adrenal cortex, while testosterone is synthesized in ALCs. In the former process, CYP17A1 mediates only 17α-hydroxylation, whereas in the latter process it mediates both 17α-hydroxylation and 17,20-lyase reaction.

Many studies have been performed to improve our understanding of the mechanism for selective regulation of these two reactions by CYP17A1^51^. Some of them have focused on the two components, POR and CYB5A, which transport electrons to CYP17A1. One study reported that POR preferentially activates the 17,20-lyase reaction^52^, while another reported that CYB5A is responsible for this activation^53^. Concordantly, a KO study has shown that *Cyb5a* is necessary for 17,20-lyase activity in ALCs^54^. Unexpectedly, however, the expression of *Por* and *Cyb5a* was not decreased in the XX/*Sry* ALCs*.* Another possible regulatory mechanism of the two reactions, phosphorylation of CYP17A1 by cAMP-dependent protein kinase, p38α, and an unknown kinase activated under serum-free condition has been shown to selectively increase 17,20-lyase activity^55–57^. Unfortunately, however, our preliminary study failed to detect the phosphorylated CYP17A1 in the XY and XX/*Sry* testes. Although we could not unveil the mechanism for the selective regulation of the CYP17A1-mediated reactions, our study revealed that XX/*Sry* ALCs could be an excellent cellular tool for future investigation of it.

In our previous study, we examined histone modifications and showed that accumulation of H3K4me3 around the upstream regions of cholesterogenic genes was reduced in XX/*Sry* Sertoli cells^6^. Considering that H3K4me3 is a mark for an active promoter, we concluded that this reduction may have led to the decreased expression of cholesterogenic genes in the XX/*Sry* Sertoli cells. Interestingly, our present study demonstrated that immediate early genes and cholesterogenic genes were differentially altered in XX/*Sry* ALCs and XX/*Sry* Sertoli cells. Comparison of whole-genome histone modifications could contribute to a deeper understanding of the mechanisms underlying cell-type-specific alteration of gene expression in XX/*Sry* mice.

## Materials and methods

### Animals

Wild-type XY C57BL/6 and XX sex-reversed transgenic mice carrying the *Hsp-Sry* transgene were used^58^. The presence of the transgene and genetic sex were confirmed via PCR with primers for *Hsp-Sry* and SX^59^ (Supplemental Table 3). SX is a single set of primers to amplify *Xlr* and *Sly* on the X- and Y-chromosome, respectively, giving distinct banding patterns after electrophoresis. We also used *Ad4BP-BAC-EGFP* mice and *mFLE-mCherry* mice^60^, in which Leydig cells and FLCs are labeled with EGFP and mCherry, respectively. *Sry* transgenic mice were crossed with *Ad4BP-BAC-EGFP;mFLE-mCherry* mice to obtain EGFP single-positive ALCs from the testes of XX/*Sry* mice. All protocols for the animal experiments were approved by the Animal Care and Use Committee of Kyushu University. All experiments were performed in accordance with the institutional guidelines.

### Cell counting and sorting

Testes were collected from eight-week-old *Ad4BP-BAC-EGFP;mFLE-mCherry* double-transgenic mice and dispersed with collagenase^30^. Total numbers of cells from XY and XX/*Sry* testes were counted using a Countess II FL (Thermo Fisher Scientific, Waltham, MA, USA). The dispersed cells were subjected to FACS using a BD FACS Aria SORP (BD Biosciences, San Jose, CA, USA) and FACS Diva software (BD Biosciences) to sort the cells into two populations based on EGFP and mCherry fluorescence (ALCs: EGFP single-positive; FLCs: EGFP/mCherry double-positive). 1,000,000 cells were analyzed to obtain the percentages of ALCs and FLCs, which were converted to the absolute numbers per testis by multiplying the total numbers of testicular cells. The EGFP single-positive ALCs were purified by performing two FACS cycles.

### Immunofluorescence analyses

Eight-week-old mice were perfused with 4% paraformaldehyde (PFA) and their testes were collected and then immersed in 4% PFA at 4 °C for 48 h. The samples were subsequently cryoprotected in 20% sucrose at 4 °C and embedded in OCT Compound (Sakura Finetek, Torrance, CA, USA). Immunofluorescence analyses were performed as described previously^11^. A rabbit antibody against HSD3B6^61^ (1:500), a chicken antibody against EGFP (ab13970, 1:1000; Abcam, Cambridge, UK), and a mouse antibody against mCherry (ab125096, 1:200; Abcam) were used as the primary antibodies. Alexa Fluor 488-labeled goat anti-chicken IgY antibody (ab150169, 1:500; Abcam), Alexa Fluor 555-labeled goat anti-mouse IgG antibody (A28180, 1:500; Life Technologies, Carlsbad, CA, USA), and Alexa Fluor 647-labeled goat anti-rabbit IgG antibody (A27040, 1:500; Life Technologies) were used as the secondary antibodies. Nuclei were stained with DAPI (4′6-diamidino-2-phenylindole; Sigma–Aldrich, St. Louis, MO, USA). Immunofluorescence was observed under a BZ-X700 microscope (Keyence, Osaka, Japan).

### In situ hybridization and immunohistochemistry

In situ hybridization was performed as previously described^62^. RIKEN FANTOM cDNA clones for *Agt* (angiotensinogen, A730059G17), *Hmgcs2* (3-hydroxy-3-methylglutaryl-coenzyme A synthase 2, 1300002P16), *Ptgds* (prostaglandin D2 synthase, 2010004I02), *Lcn2* (lipocalin-2, 2G530015N18), and *Sepp1* (selenoprotein P, plasma, 1; I920052L16) were purchased (DNAFORM, Yokohama, Japan). Digoxigenin-labeled riboprobes for these genes were used (Roche, Basel, Switzerland).

### qRT-PCR

qRT-PCR was performed as previously described^63^ and conducted following the MIQE guidelines^64^. In brief, total RNA was isolated from the sorted cells or tissues using RNeasy Micro Kit or RNeasy Mini Kit (Qiagen, Hilden, Germany) and 50 ng of total RNA was reverse-transcribed to cDNA using random hexamers and M-MLV Reverse Transcriptase (Thermo Fisher Scientific). RNA integrity numbers (RINs) of all samples were confirmed to be higher than 7.5 using a Bioanalyzer (Agilent Technologies, Santa Clara, CA, USA). qRT-PCR was performed using a CFX96 Real-Time PCR Detection System (Bio-Rad, Hercules, CA, USA) with the SYBR Select Master Mix (Applied Biosystems, Foster City, CA, USA). Gene expression was determined using the standard curve method. The correlation coefficients (R^2^) for the standard curves were higher than 0.99. Gene expression levels were normalized to those of *Actb* (β-actin). The primers used for the PCR are listed in Supplemental Table 3.

### mRNA sequencing

mRNA sequencing was performed as described previously^30^. Briefly, poly(A) RNA content was isolated from total RNA (10 ng per sample) prepared from sorted XY and XX/*Sry* ALCs using the NEBNext Poly(A) mRNA Magnetic Isolation Module (New England Biolabs, Ipswich, MA, USA). Sequence libraries were constructed using NEBNext Ultra II Directional RNA Library Prep Kit for Illumina (New England Biolabs) and NEBNext Multiplex Oligo for Illumina (Dual Index Primers Set 1; New England Biolabs). cDNA libraries were sequenced using a NovaSeq 6000 (51-bp pair-end; Illumina, San Diego, CA, USA).

### Data processing

The FastQ files were mapped using STAR software^65^ (version 2.7.0a; standard option) to the mouse reference genome (UCSC mm10) and the genome annotation (modified to integrate the EGFP and mCherry transgenes) was downloaded from the UCSC Genome Browser. Bam files were generated using SamTools^66^ (version 0.3.3). Quality control, mapping, read count, and CPM (counts per million mapped reads) were computed using featureCounts^67^ (version 1.6.4; option ‘-O -p’), edgeR^68^ (version 3.20.9), and an in-house pipeline. MicroRNA and small nucleolar RNA genes were excluded from the analyses. Gene expression data are presented as CPM. Mean values for biological replicates (n = 3) were calculated, and genes with CPM values < 20 in both XY and XX/*Sry* ALCs were removed. Differentially expressed genes were identified based on fold change and subjected to Gene Ontology (GO) analyses using DAVID. The significantly enriched biological process GO terms with *p*-values < 0.001 were visualized in two-dimensional plots using REVIGO^69^. Fold changes in gene expression levels (XX/*Sry* vs. XY) for Sertoli cells were also calculated using the transcriptome data in our previous study^6^ (accession number: DRA004090). When comparing whole gene expression changes in the two types of cells, a pseudo-count of 10 was added to the CPM values before the fold changes were calculated.

### Measurement of intratesticular sex steroids

Testes obtained from eight-week-old XY and XX/*Sry* mice were lyophilized using a Vacuum Centrifugal Evaporator (CVE-2000; EYELA, Tokyo, Japan) and stored at − 80 °C until later use. Gas chromatography–mass spectrometry steroid profiling was performed using a Shimadzu GC 2010 Plus gas chromatograph coupled to a triple-quadrupole GCMS-TQ8050 (Shimadzu Corporation, Kyoto, Japan) as previously described^70^. Quantitative results were based on absolute quantities of steroid molecules per testis, and their metabolic ratios were also calculated to express their corresponding enzymatic activities.

### Statistical analysis

At least three biologically independent samples were used in all experiments. Data are presented as mean ± SEM. Differences between XY and XX/*Sry* cells or testes were examined using two-tailed Student’s *t*-tests or Mann–Whitney *U* tests, and statistical significance was inferred at *p* < 0.05. All statistical analyses were performed using R software version 3.4.3 (https://www.r-project.org).

## Supplementary Information

Supplementary Information 1.

Supplementary Information 2.

Supplementary Information 3.

Supplementary Information 4.

## Data Availability

The transcriptome data have been deposited in DDBJ under the accession number DRA009797 and DRA010792.
