# Supplementary material for: Gene expression and functional abnormalities in XX/Sry Leydig cells
Source: Sci Rep. 2021 Jan 12;11:719. doi: 10.1038/s41598-020-80741-z (PMC7804417; doi:10.1038/s41598-020-80741-z)
Supplement: Supplementary file 1 — Supplementary Information 1. [file 41598_2020_80741_MOESM1_ESM.pdf]

Supplemental Figures

Gene expression and functional abnormalities in XX/*Sry* Leydig cells

Shogo Yanai, Takashi Baba, Kai Inui, Kanako Miyabayashi, Soyun Han, Miki Inoue, Fumiya Takahashi, Yoshiakira Kanai, Yasuyuki Ohkawa, Man Ho Choi, Ken-ichirou Morohashi

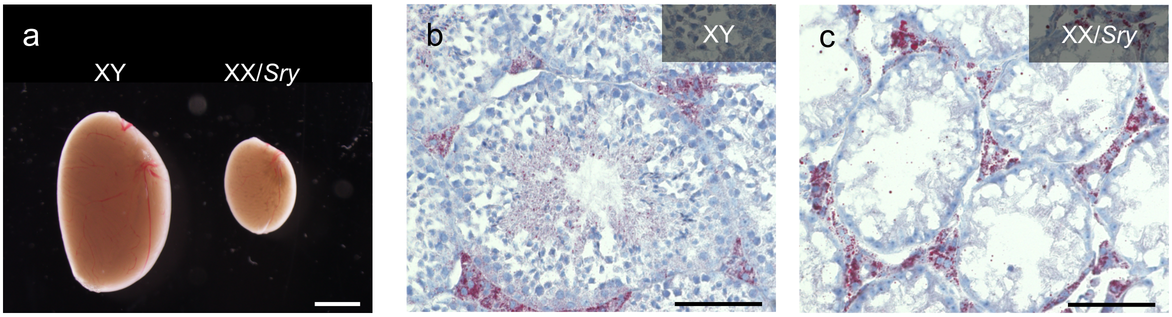

Supplemental Fig. 1, Structural differences between XY and XX/Sry testes. ***a***, Whole view of XY and XX/*Sry* testes of eight-week-old mice are shown. Scale bar = 2 mm. ***b*** and ***c***, XY and XX/*Sry* testes of eight-week-old mice were stained with Oil red O and hematoxylin. Scale bars = 50 µm.

Supplemental Fig. 1, Yanai *et al*.,

a

	Total reads	Uniquely mapped reads	Reads mapped to multiple loci
XY ALC1	12232191	11022073 (90.1%)	924025 (7.6%)
XY ALC2	22898338	20799117 (90.8%)	1653423 (7.2%)
XY ALC3	19385200	17570984 (90.6%)	1340047 (6.9%)
XX/*Sry* ALC1	13613624	12188410 (89.5%)	973661 (7.2%)
XX/*Sry* ALC2	14040206	12725129 (90.6%)	1052764 (7.5%)
XX/*Sry* ALC3	21150652	19238771 (91.0%)	1478237 (7.0%)

b

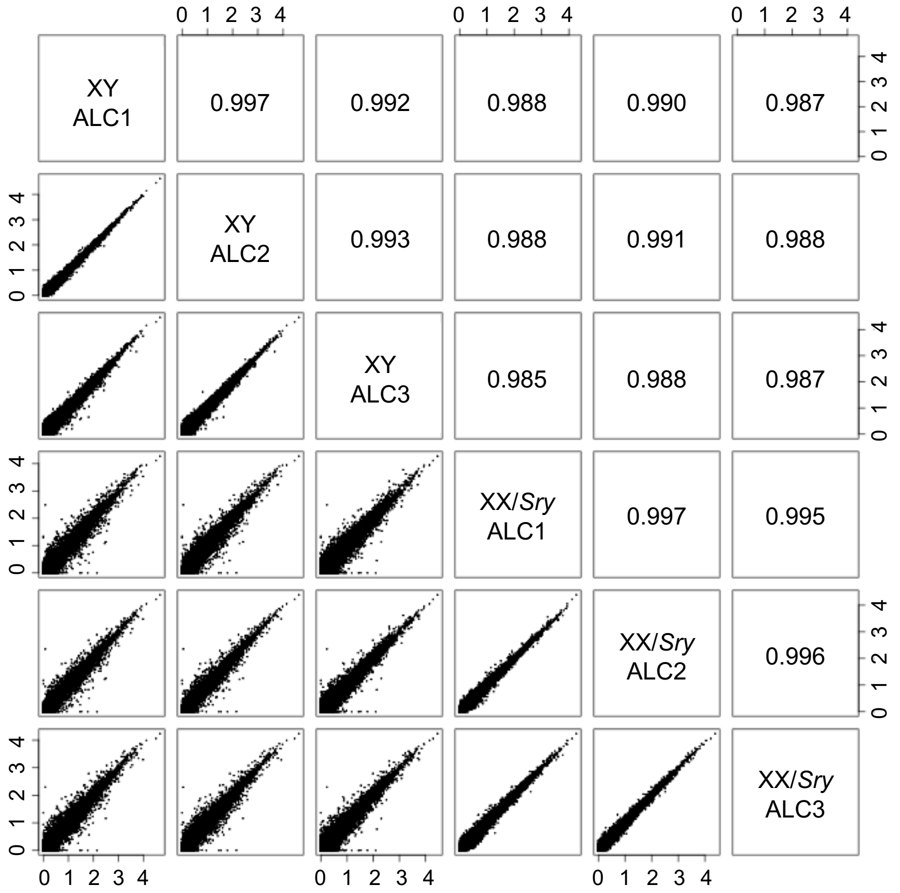

Supplemental Fig. 2, Qualities of transcriptome data. ***a***, Transcriptomes were obtained from three biologically independent ALCs each for XY and XX/*Sry* testes. Total reads, uniquely mapped reads, and reads mapped to multiple loci are summarized for each transcriptome study. ***b***, Gene expression was compared pairwise between samples. Scatter plots (log_{10} scale) are shown upper the diagonal and correlation coefficients below the diagonal.

a Sertoli cells

	Gene	CPM XY	CPM XX/*Sry*	Fold change
PDGF	*Pdgfa*	0.8	5.2	3.44
	Pdgfb	1.2	0.5	0.71
	Pdgfc	55.9	106.3	1.88
	Pdgfd	0.2	0.8	1.46
Hedgehog	*Dhh*	234.4	277.8	1.18
	Ihh	0.1	0.3	1.14
	Shh	0.0	0.0	1.00
TGFβ	*Tgfb1*	58.9	47.9	0.82
	Tgfb2	0.5	2.8	2.55
	Tgfb3	127.8	121.8	0.95
FGF	*Fgf2*	0.0	0.0	1.00
Activin	*Inha*	1321.4	1672.8	1.27
	Inhba	0.1	0.7	1.52
	Inhbb	461.2	337.1	0.73
IGF1	*Igf1*	1.1	5.9	3.27

b ALCs

	Gene	CPM XY	CPM XX/*Sry*	Fold change
PDGF	*Pdgfra*	247.9	431.0	1.74
	Pdgfrb	0.6	2.4	2.12
Hedgehog	*Ptch1*	75.2	67.8	0.90
	Ptch2	0.1	1.1	1.87
	Smo	96.0	100.4	1.04
TGFβ	*Tgfbr1*	43.1	39.3	0.91
	Tgfbr2	2.3	3.9	1.50
	Tgfbr3	406.8	548.7	1.35
FGF	*Fgfr1*	55.1	76.7	1.38
	Fgfr2	180.4	173.8	0.96
	Fgfr3	0.1	0.2	1.12
	Fgfr4	75.1	53.0	0.71
Activin	*Acvr1*	150.9	156.2	1.03
	Acvr1b	6.0	7.4	1.19
	Acvr1c	10.0	18.1	1.73
	Acvr2a	28.8	32.4	1.12
	Acvr2b	0.8	1.2	1.23
IGF1	*Igf1r*	275.8	301.9	1.09

Supplemental Fig. 3, Expression of genes related to paracrine factors. ***a***, Expression of paracrine factors involved in the differentiation of ALCs was extracted from the transcriptome datasets for the XY and XX/*Sry* Sertoli cells. Increased and decreased gene expression in the XX/*Sry* Sertoli cells is indicated in red and blue, respectively, with deeper shading for larger differences. ***b***, Expression of receptors for the paracrine factors was extracted from the transcriptome datasets for the XY and XX/*Sry* ALCs. CPMs are means of biological triplicates. Increased and decreased gene expression in the XX/*Sry* ALCs is indicated in red and blue, respectively, with deeper shading for larger differences.

Supplemental Fig. 3, Yanai *et al.*,

a

Gene	CPM		Fold change	Regulation	Target	Reference
	XY	XX/*Sry*				
Cebpa	80	95	1.19	Activation	*Star*	1
Cebpb	122	55	0.45	Activation	*Star*<br>*CYP11A1*<br>*HSD3B2*	1,2
Creb1	28	35	1.26	Activation	*Star*<br>*CYP11A1*	1,3
Fos	2959	1877	0.63	Activation	*Star*<br>*CYP11A1*	1,4
Gata4	200	203	1.02	Activation	*HSD3B1*<br>*Cyp17a1*	5,6
Hif1a	54	61	1.12	Activation	*Hsd3b1*	7
Jun	1590	1389	0.87	Activation	*Star*	1
Nfkb1	55	54	0.99	Suppression	*Cyp17a1*	8
Nfkb2	27	25	0.92	Suppression	*Cyp17a1*	8
Nr3b3/Esrr3	24	23	0.96	Activation	*Cyp17a1*	9
Nr3c1/gr	44	67	1.52	Suppression	*Star*	10
Nr4a1/Nur77	133	123	0.92	Activation	*HSD3B2*<br>*Star*<br>*Cyp17a1*	10,11,12
Sp1	69	90	1.31	Activation	*Star*<br>*CYP11A1*<br>*Cyp17a1*	1,6,13
Srebf1	135	180	1.33	Activation	*Star*	1
Yy1	67	71	1.05	Activation	*HSD3B2*	14

Supplemental Fig. 4, Expression of genes encoding transcription factors. ***a***, Expression of transcription factors involved in the regulation of steroidogenesis was extracted from the transcriptome datasets for the XY and XX/Sry ALCs. CPMs are means of biological triplicates. Increased and decreased gene expression in the XX/*Sry* ALCs is indicated in red and blue, respectively, with deeper shading for larger differences.

Supplemental Fig. 4, Yanai *et al*.,

References

1. Manna, P. R., Wang, X.-J. & Stocco, D. M. Involvement of multiple transcription factors in the regulation of steroidogenic acute regulatory protein gene expression. *Steroids* **68**, 1125–1134 (2003).
2. Mizutani, T. *et al.* C/EBPβ (CCAAT/enhancer-binding protein β) mediates progesterone production through transcriptional regulation in co-operation with SF-1 (steroidogenic factor-1). *Biochem. J.* **460**, 459–471 (2014).
3. Morohashi, K. *et al.* Activation of CYP11A and CYP11B gene promoters by the steroidogenic cell-specific transcription factor, Ad4BP. *Mol. Endocrinol.* **7**, 1196–1204 (1993).
4. Guo, I. C., Huang, C. Y., Wang C. K. & Chung, B. C. Activating Protein-1 Cooperates with Steroidogenic Factor-1 to Regulate 3′,5′-Cyclic Adenosine 5′-Monophosphate-Dependent Human CYP11A1 Transcription in Vitro and in Vivo. *Endocrinology* **148**, 1804–1812 (2007).
5. Bergeron, F., Nadeau, G. & Viger, R. S. GATA4 knockdown in MA-10 Leydig cells identifies multiple target genes in the steroidogenic pathway. *Reproduction* **149**, 245–257 (2015).
6. Flück, C. E. & Miller, W. L. GATA-4 and GATA-6 modulate tissue-specific transcription of the human gene for P450c17 by direct interaction with Sp1. *Mol. Endocrinol.* **18**, 1144–1157 (2004).
7. Lysiak, J. J. *et al.* Hypoxia-inducible factor-1alpha is constitutively expressed in murine Leydig cells and regulates 3beta-hydroxysteroid dehydrogenase type 1 promoter activity. *J. Androl.* **30**, 146–156 (2009).
8. Hong, C. Y. *et al.* Molecular mechanism of suppression of testicular steroidogenesis by proinflammatory cytokine tumor necrosis factor alpha. *Mol. Cell. Biol.* **24**, 2593–2604 (2004).
9. Park, E. *et al.* Estrogen receptor-related receptor γ regulates testicular steroidogenesis through direct and indirect regulation of steroidogenic gene expression. *Mol. Cell. Endocrinol.* **452**, 15–24 (2017).
10. Martin, L. J. & Tremblay, J. J. Glucocorticoids antagonize cAMP-induced Star transcription in Leydig cells through the orphan nuclear receptor NR4A1. *J. Mol. Endocrinol.* **41**, 165–175 (2008).
11. Havelock, J. C. *et al.* The NGFI-B family of transcription factors regulates expression of 3beta-hydroxysteroid dehydrogenase type 2 in the human ovary. *Mol. Hum. Reprod.* **11**, 79–85 (2005).
12. Martin, L. J. & Tremblay, J. J. Nuclear receptors in Leydig cell gene expression and function. *Biol. Reprod.* **83**, 3–14 (2010).
13. Guo, I. C., Hu, M. C. & Chung, B. C. Transcriptional Regulation of *CYP11A1*. *J. Biomed. Sci.* **10**, 593–598 (2003).
14. Foti, D. M. & Reichardt, J. K. YY1 binding within the human HSD3B2 gene intron 1 is required for maximal basal promoter activity: identification of YY1 as the 3beta1-A factor. *J. Mol. Endocrinol.* **33**, 99–119 (2004).
